# Genetic interactions regulating seed phytate and oligosaccharides in soybean (*Glycine max* L.)

**DOI:** 10.1371/journal.pone.0235120

**Published:** 2020-06-25

**Authors:** Neelam R. Redekar, Natasha M. Glover, Ruslan M. Biyashev, Bo-Keun Ha, Victor Raboy, M. A. Saghai Maroof

**Affiliations:** 1 School of Plant and Environmental Sciences, Virginia Tech, Blacksburg, Virginia, United States of America; 2 Institute of Plant Breeding, Genetics & Genomics, University of Georgia, Athens, Georgia, United States of America; 3 National Small Grains Germplasm Center, USDA-ARS, Aberdeen, Idaho, United States of America; University of Guelph, CANADA

## Abstract

Two low-phytate soybean (*Glycine max* (L.) Merr.) mutant lines– V99-5089 (*mips* mutation on chromosome 11) and CX-1834 (*mrp-l* and *mrp-n* mutations on chromosomes 19 and 3, respectively) have proven to be valuable resources for breeding of low-phytate, high-sucrose, and low-raffinosaccharide soybeans, traits that are highly desirable from a nutritional and environmental standpoint. A recombinant inbred population derived from the cross CX1834 x V99-5089 provides an opportunity to study the effect of different combinations of these three mutations on soybean phytate and oligosaccharides levels. Of the 173 recombinant inbred lines tested, 163 lines were homozygous for various combinations of MIPS and two MRP loci alleles. These individuals were grouped into eight genotypic classes based on the combination of SNP alleles at the three mutant loci. The two genotypic classes that were homozygous *mrp-l/mrp-n* and either homozygous wild-type or mutant at the *mips* locus (MIPS/*mrp-l/mrp-n* or *mips/mrp-l/mrp-n*) displayed relatively similar ~55% reductions in seed phytate, 6.94 mg g ^-1^ and 6.70 mg g^-1^ respectively, as compared with 15.2 mg g^-1^ in the wild-type MIPS/MRP-L/MRP-N seed. Therefore, in the presence of the double mutant *mrp-l/mrp-n*, the *mips* mutation did not cause a substantially greater decrease in seed phytate level. However, the nutritionally-desirable high-sucrose/low-stachyose/low-raffinose seed phenotype originally observed in soybeans homozygous for the *mips* allele was reversed in the presence of *mrp-l/mrp-n* mutations: homozygous *mips/mrp-l/mrp-n* seed displayed low-sucrose (7.70%), high-stachyose (4.18%), and the highest observed raffinose (0.94%) contents per gram of dry seed. Perhaps the block in phytic acid transport from its cytoplasmic synthesis site to its storage site, conditioned by *mrp-l/mrp-n*, alters myo-inositol flux in *mips* seeds in a way that restores to wild-type levels the *mips* conditioned reductions in raffinosaccharides. Overall this study determined the combinatorial effects of three low phytic acid causing mutations on regulation of seed phytate and oligosaccharides in soybean.

## Introduction

Seed metabolite reserves are crucial for seed protection during dormancy, seed germination and development into a seedling. Sucrose (disaccharide), raffinose (trisaccharide), and stachyose (tetrasaccharide) are the three main forms of carbohydrates found in soybean [*Glycine max* (L.) Merr.] seeds. Sucrose forms the major portion of the total seed carbohydrate, ranging from 5–7% of the seed dry weight [1]. Raffinose and stachyose, which make up the raffinose family oligosaccharides (RFOs) could range from 3–4% and 1% of the seed dry weight, respectively [1]. These RFOs are also involved in transporting photo-assimilates from the leaves to the phloem [2], serving as storage carbohydrates and cryoprotectants to promote freezing-tolerance [3, 4], or desiccation tolerance and longevity of seeds [5]. Seed RFOs are quickly broken down during early germination to provide the seedling with energy and substrates for growth [6]. In addition to carbohydrates, seeds also store phosphorus (P) in the form of “phytate” salts of phytic acid (inositol hexakisphophate (IP6)). In major cereal and legume crops, seeds normally contain between 3.0 and 8.0 mg of P per gram of dry weight, and out of this total P, 65–75% is phytic acid [7].

Interaction between phytic acid and RFO biosynthesis pathways supplies and regulates the levels of sucrose, raffinose, stachyose, phytate, and inositol in soybean seeds. During phytic acid biosynthesis, myo-inositol phosphate synthase (MIPS, EC 5.5.1.4) catalyzes the conversion of glucose-6-phosphate to inositol-3-phosphate, which is the sole synthetic source of the *myo*-inositol backbone [8]. Inositol-3-phosphate is then dephosphorylated to form *myo*-inositol by inositol monophosphatase enzyme. Free *myo*-inositol is then utilized in two ways relevant to this study; to produce phytic acid in a multi-step phosphorylation process and to produce galactinol from UDP-D-galactose by galactinol synthase (EC 2.4.1.123) [8]. Both the phytic acid and RFO pathways utilize free *myo*-inositol as an intermediate, which links the phytate and RFOs biochemical pathways. Suppression of MIPS gene expression with RNAi in potato (*Solanum tuberosum* L.) was shown to drastically reduce levels of inositol, galactinol, and raffinose [9]. Other studies have also shown a direct correlation between myo-inositol and RFOs and galactinol levels in soybean [10], pea (*Pisum sativum* L.) and barley (*Hordeum vulgare* L.) [11]. Together, these studies indicate that complex regulatory pathways play a role in maintaining substrates such as *myo*-inositol, sucrose, phytate, and RFOs.

Both phytic acid and RFO levels have become targets for manipulation in recent years in order to improve soybean seed quality [12]. RFOs are indigestible in monogastric animals and supply little metabolizable energy because these animals do not synthesize enough of the enzyme α-galactosidase to digest these RFOs [13]. Although RFOs can provide a beneficial probiotic function [14], they may also cause gastric discomfort, and flatulence [15, 16]. Because phytic acid chelates mineral cations including calcium, iron, and zinc, these mixed salts are often excreted by non-ruminant animals such as humans, swine, poultry, and fish [17]. Excretion of phytic acid can cause water pollution due to the excess P in animal waste that has the potential to run off into bodies of water [18]. Animal feeding trials based on barley, corn, or soybean containing low levels of oligosaccharide and/or phytic acid were found to increase the metabolizable energy and available P to animals, satisfying more of their dietary requirement [19–25]. Therefore, due to the indigestibility of phytate and RFOs and the undesirable environmental consequences, low-phytate and low-RFO varieties of soybeans are desired [7, 21].

Several attempts have been made to produce soybeans with low-phytate and RFO levels via random mutagenesis or traditional breeding methods. These mutations were later found to exist in the genes involved in regulating phytate/RFO pathway such as MIPS [10, 26–30], multidrug resistance-associated protein (MRP) ATP-binding cassette (ABC) transporters [31–36], and *myo*-inositol kinase (EC 2.7.1.64) [37]. The role of MRP transporters in phytate accumulation is not entirely clear, but Panzeri et al. [33] hypothesized that since phytate cannot be properly stored in the vacuole in low-phytate lines with defective MRPs, phytase enzymes in the cytoplasm break it down into its precursor, *myo*-inositol. This could cause negative feedback regulation, leading to lower levels of phytate. Despite the eco-friendly and pro-nutritional nature of these low-phytate and low-RFO soybeans, such genetic material exhibits poor seed vigor and reduced germination and emergence rates.

Several studies have been conducted to examine agronomic traits in low-phytate (‘CX-1834-1-6’ and ‘V99-5089’) and low-RFO (PI 200508 and V99-5089) soybeans. For example, Oltmans et al. [38] investigated multiple agronomic and seed traits in three CX-1834-1-6-derived (hereafter, CX1834) soybean populations across three environments [38]. Only seedling emergence, phytate P, and inorganic P were found significantly different between selected low-phytate and normal-phytate lines from each of the three populations [38]. Boehm et al. [39] examined seed yield and field emergence of two CX1834-derived *lpa* soybean lines across six environments and concluded that *lpa* crops can produce seed yields statistically equivalent to high-yielding control cultivars with no germination issues [39]. Bilyeu and Wiebold [40] examined stability of the PI 200508-derived soybean seed carbohydrate profile across nine environments and found that soybean seed sucrose content was more variable across environments than stachyose [40]. They also observed strong correlation between cooler temperatures during the later pod-filling stages and increased sucrose and decreased stachyose contents [40]. Maupin et al. [41] examined agronomic and seed traits of a V99-5089-derived recombinant inbred line (RIL) population over two years in three environments and found genotype by environment interactions to be key determinants of seed inorganic P, but not sugar content in low-phytate mutants [41]. They also observed significant negative correlation between Pi concentration and seed emergence [41]. Understanding genetic and molecular bases of the *lpa* trait is required to comprehend regulation of seed P and carbohydrates, address the low emergence problem, and accomplish the development of high yielding *lpa* crops.

The current study is focused on understanding the impact of combining *lpa* causing mutations in a MIPS gene (in *lpa* line V99-5089) [42] and two MRP genes (in *lpa* line CX1834) [31, 43] on seed phytate and oligosaccharides in soybean. Seeds of V99-5089 exhibit a low-phytate, low-stachyose, and high-sucrose phenotype; whereas, seeds of CX1834, exhibit low-phytate, normal-stachyose, and normal-sucrose phenotype. A recombinant inbred line population was developed from a cross of V99-5089 x CX1834 and studied the effects of different combinations of the mutations of MIPS located on chromosome 11 (Linkage group (LG) B1) and the MRPs on chr. 19 (LG L) and chr. 3 (LG N) on phytate, and oligosaccharide. The V99-5089 x CX1834 RIL population has provided an opportunity to determine the effects of all possible combinations of these three mutations on seed phenotypes of interest. It was hypothesized that combining all three mutations would lead to low-phytate, low-RFOs, and normal-sucrose.

## Materials and methods

### Genetic materials

#### Source of parental lines

CX1834 is a low-phytate line derived from a cross between ‘Athow,’ a normal-phytate soybean cultivar, and M153-1-4-6-14, a low-phytate mutant [43]. CX1834 carries two-point mutations, one in an MRP gene (Glyma.03G167800, MRP-N) on chr. 3 (LG N), and one in an MRP gene (Glyma.19G169000, MRP-L) on chr. 19 (LG L) [31, 34]. V99-5089 is a Virginia Tech experimental line with low-phytate, low-RFOs, and high-sucrose due to mutation in MIPS gene (Glyma.11G238800, MIPS) on chr. 11 (LG B1) [42]. Both parental lines (CX1834 and V99-5089) were planted in the field in 2008 along with the rest of the F_8_ population. One bulk sample for each parent was collected and assayed for phytate and oligosaccharide content (nine times for V99-5089 and seven times for CX1834).

#### Recombinant inbred line (RIL) population

The CX1834 x V99-5089 RILs population was planted at Kentland Farm, Virginia in 2006, 2007, 2008, 2009, and 2010, and from which F_6_, F_7_, F_8_, F_9_, and F_10_ generation seeds were harvested, respectively. Total of 173 RILs was used for marker and phenotypic data collection (F_8_ population for seed phytate, F_6_ and F_8_ populations for oligosaccharide content). The F_8_ population was tested for the presence of *lpa* causing mutations on the MIPS gene (*mips* on chr. 11) and the MRP genes (*mrp-l* on chr. 19 and *mrp-n* on chr. 3) (see below Marker Data Collection).

#### Low-phytate causing mutations

The three-point mutations controlling phytate and oligosaccharide content occur in the MIPS gene on chr. 11 (LG B1) and in the MRP ABC transporter genes on chr. 19 (LG L) and chr. 3 (LG N). Soybean line V99-5089 has a ‘C’ to a ‘G’ mutation (henceforth ‘*mips*’) in the MIPS gene that is responsible for low-stachyose, high-sucrose, and low-phytate content in seeds [42]. Soybean line CX1834 has two *lpa* causing mutations–an ‘A’ to a ‘T’ mutation (henceforth ‘*mrp-n*’) on chr. 3 (LG N), and a ‘G’ to ‘A’ mutation on chr. 19 (LG L) (henceforth ‘*mrp-l*’) in two different MRP ABC transporter genes [31, 34]. The genotypes at these three mutation sites were represented as “MIPS/MRP-L/MRP-N”, where the mutant allele was indicated by an italicized gene name in lower case letters and the wild-type allele was indicated by its gene name in uppercase letters. For example, the “*mips*/MRP-L/MRP-N” genotypic class has only one mutation in MIPS gene on chr. 11 (LG B1), similar to the first parent V99-5089; whereas, “MIPS/*mrp-l*/*mrp-n*” genotypic class contains mutations in two MRP genes on chr. 19 (LG L) and chr. 3 (LG N), similar to the second parent CX1834 (see also Table 1 and its footnote for allele and genotype designations).

**Table 1:** Genotypic classes of the homozygous RILs of V99-5089 x CX-1834 population.

**Genotypic class** ^a^	**SNP alleles**^b^			**N**
	MIPS	MRP-L	MRP-N	
*mips*/MRP-L/MRP-N	*g*	G	A	15
*mips*/MRP-L/*mrp-n*	*g*	G	*t*	14
*mips/mrp-l*/MRP-N	*g*	*a*	A	20
*mips*/*mrp-l/mrp-n*	*g*	*a*	*t*	14
MIPS/MRP-L/MRP-N	C	G	A	29
MIPS/MRP-L/*mrp-n*	C	G	*t*	24
MIPS/*mrp-l/*MRP-N	C	*a*	A	30
MIPS/*mrp-l/mrp-n*	C	*a*	*t*	17

^a^ Genotypic class are designated in order of MIPS allele on chr. 11 (LG B1)/MRP-L allele on chr. 19 (LG L)/MRP-N allele on chr. 3 (LG N). Lower case, italicized letters indicate mutant alleles and upper-case letters indicate wild-type alleles.

^b^ For MIPS gene (Glyma.11G238800) on chr. 11, the mutation is ‘G’ and the wild type is ‘C’. For the MRP-L gene (Glyma.19G169000) on chr. 19, the mutation is ‘A’, and wild type is ‘G’. For the MRP-N gene (Glyma.03G167800) on chr. 3, the mutation is ‘T’ and wild type is ‘A’.

### Marker data collection

Approximately 30 F_8_ seeds for each of 173 RILs were planted in the greenhouse. Trifoliate leaf tissue from 10 to 16 plants for each RIL were pooled for DNA extraction, performed according to Yu et al. [44]. The physical locations of the MRP mutations in CX1834 on chr. 19 and chr. 3 [31, 34] as well as the MIPS mutation in V99-5089 were used to design primers for single nucleotide polymorphism (SNP) genotyping. The RIL genotyping was performed at the University of Georgia, using the SimpleProbe melting curve analysis (TIB MOLBIOL, Adelphia, NJ, USA). Three sets of SNP assays were designated as SNP-L, SNP-N, and SNP-B1 for chr. 19, chr. 3, and chr. 11, respectively. Every assay consisted of forward and reverse primers and a fluorescein tagged probe for SNP detection. The SNP-L forward primer sequence was 5’-CTGAATTTAAATGCACGTC-3’, the reverse primer sequence was 5’-TGTGAAGCTGAGGTTAG-3’, and the SimpleProbe primer sequence was 5‘-TTGGCTGTACTGATA**XI**AATT**C**TCTCAATAG–Phosphate-3’. The SNP-N forward primer sequence was 5’-CCTGGAGGCATCTGTTATGAC-3’, the reverse primer sequence was 5’-CTGCCATGTATGAAAGAT-3’, and the SimpleProbe primer sequence was 5’-CAAGCTGTT**XI**TCTTTC**A**CGATCGTT—Phosphate-3’. The SNP-B1 forward primer sequence was 5’-AACAATGATGGTATGAATCTTTCG-3’, the reverse primer sequence was 5’-CCTGACAAGAGAAAGAAACAGA-3’, and the SimpleProbe primer sequence was 5‘-gTgAACA**XI**TCCAgAC**C**ATgTTgTTgTT-Phosphate-3’. “XI” was the internal SimpleProbe label, and the position of the SNP in the probes is underlined and bolded. Fluorescein was used to internally label the SimpleProbes.

DNA from the F_8_ CX1834 x V99-5089 RILs was used for genotyping the SNP mutations on all three chromosomes. A LightCycler 480 (Roche Applied Science, Indianapolis, IN, USA) was used for PCR reactions, with a total volume of 3 µL per well, using the protocol for asymmetrical PCR. Each PCR reaction consisted of 20–30 ng of genomic DNA, 0.5 μM of limiting primer, 1.0 μM of excess primer, 0.2 μM of SimpleProbe, 3.0 mM MgCl_2_, and 0.5X of LightCycler 480 Genotyping Master mix (Roche Applied Science, Indianapolis, IN, USA).

Fifty PCR cycles were performed with 10 s of denaturation at 95°C, 15 s of annealing at 60°C, and 20 s of extension at 72°C. A final melting cycle was performed at 95°C for 2 min, 40°C for 2 min, and increasing the temperature to 85°C, at this point using continuous fluorescent acquisition, and followed by a decrease in temperature to 40°C. To generate melting curves for each sample, the fluorescence signal (*F*) was plotted against temperature (*T*) in real time. Negative derivative curves of fluorescence with respect to temperature (-d*F*/d*T*) were produced by the LightCycler Data Analysis software (Roche Diagnostics, Indianapolis, IN, USA). In order to confirm the SNP marker data for DNA samples collected from *mips/mrp-l/mrp-n* RILs (n = 14) from this population, the samples were sequenced for the mutations on MIPS gene (Glyma.11G238800) on chr. 11 and two MRP genes (Glyma.19G169000 and Glyma.03G167800) on chr. 19 and chr. 3 with the procedure according to Saghai Maroof et al. [34].

### Phytate data collection

Approximately 75 seeds from each of the 173 RILs were ground to fine powder for measuring phytate levels in seeds using a modified colorimetric method [45, 46]. Briefly, 0.5 g of soybean powder was weighed into 14 mL falcon tubes. To each tube, 10 mL of 0.65 M HCl was added, and the tubes were vortexed and put on a shaker overnight (220 rpm at room temperature). The samples were then centrifuged at 3300 rpm at 10°C for 15 min, 500 µL of the supernatant was transferred to a microcentrifuge tube and mixed with 500 µL of 20% NaCl solution. After 2-hour precipitation of the crude extract, the samples were centrifuged at 13200 rpm for 15 min. From each sample, 120 µL of the supernatant was added to 2.88 mL of ddH_2_O (25 times dilution), and an additional 1 mL of Wade’s Reagent (0.03% FeCl_3_·6H_2_O + 0.3% sulfosalicylic acid) was added for color development. Next, the samples were centrifuged at 3500 rpm for 10 min at 10°C, and absorbance was measured on a Beckman Coulter DU 800 Spectrophotometer (Fullerton, CA) at 500 nm to determine phytate content. Phytate concentrations of the samples were calculated using a calibration curve consisting of eight standards (0, 1.12, 2.24, 3.36, 5.6, 7.84, 8.96, and 11.2 ppm phytate). These standards also contained HCl and NaCl to decrease matrix effects.

### Oligosaccharide data collection

Oligosaccharide data was collected for 173 RILs from F_6_ and F_8_ generations. The oligosaccharide data was also collected for a subset of F_10_ seeds (14 RILs with *mips/mrp-l/mrp-n* genotype). Determination of sugar content in soybean seeds by high performance liquid chromatography (HPLC) was based on the procedure originally described by Cicek et al.[47], with modifications. Briefly, about 1 g of ground sample from each of the 173 RILs in the population was weighed into 12 mL centrifuge tubes, and 10 mL of ddH_2_O were added to each sample. The samples were mixed by vortexing and shaking at 200 rpm for 20 min. Next, the samples were centrifuged for 10 min at 4000 rpm. From these centrifuged samples, 0.5 mL of supernatant was transferred into another 1.5 mL centrifuge tube, and 0.7 mL of 100% acetonitrile was added to each tube. The contents of the tube were mixed and allowed to sit at room temperature for 2 hrs. Next, 1.0 mL of the supernatant was taken from each sample and was dried at 80°C under airflow. The samples were then dissolved with 0.5 mL of 65% acetonitrile, centrifuged at 13,000 rpm for 10 min, and transferred to HPLC vials for analysis.

Sucrose, raffinose, and stachyose were measured with an Agilent 1200 high performance liquid chromatograph with a differential refractrometer detector (refractive index detector) (Santa Clara, CA). A Supelco apHera NH_2_ analytical column (4.6 x 250 mm, 5 µm) was used for separation, along with a Supelco apHera NH_2_ guard column (1 cm x 4.6 mm, 5 µm). All reagents were prepared using ddH_2_O, and chemicals were all analytical or HPLC grade. The mobile phase, 65% acetonitrile, was made by combining 65 mL of 100% acetonitrile and 35 mL of ddH_2_O and filtering through a 0.45 µm filter. The elution method was 65% acetonitrile with a flow rate of 1 mL min^-1^, and 10 µL from each sample was used for injection.

### Statistical analyses

For seed phytate and oligosaccharide data, we were only interested in the homozygous lines. Thus, 10 RILs that were heterozygous at either of the MIPS, MRP-L, or MRP-N mutation sites were not included in this study. All statistical analyses including descriptive statistics and probability density distributions of the seed phenotype data for 163 RILs were performed with the R statistical program. Data normality was tested with the Shapiro-Wilk normality test and was found not normally distributed. Kruskal-Wallis rank sum test was used to determine if there are statistically significant differences in seed phenotype data between the eight genotypic classes. A Pairwise Wilcox test was used to compare group means at the significance level of 0.01 (for oligosaccharides data) and 0.05 (for phytate data).

## Results

### SNP genotyping reveals eight genotypic classes of the V99-5089 x CX1834 RIL population

SNP alleles at these three mutation sites on chr. 11, 19, and 3 that control phytate and/or oligosaccharide content were collected for 173 RILs of the V99-5089 x CX1834 population (F_8_ generation). Ten RILs were heterozygous at one, two or all three-mutation sites, while the remaining 163 RILs were homozygous at all three mutation sites. The 163 homozygous RILs could be divided into eight genotypic classes, each representing different combinations of the mutations on chr. 11, 19, and 3: *mips*/MRP-L/MRP-N, *mips*/MRP-L/*mrp-n*, *mips*/*mrp-l*/MRP-N, *mips*/*mrp-l*/*mrp-n*, MIPS/*mrp-l*/*mrp-n*, MIPS/MRP-L/*mrp-n*, MIPS/*mrp-l*/MRP-N, and MIPS/MRP-L/MRP-N (**Table 1**). The observed segregation ratio for these genotypic classes was significantly different from the expected 1:1:1:1:1:1:1:1 segregation ratio (χ^2^ = 15.28, df = 7, p-value = 0.0396, α = 0.05). The number of RILs within a genotypic class ranged from 14 individuals for the *mips*/*mrp-l*/*mrp-n* and *mips*/MRP-L/*mrp-n* classes to 30 individuals for the MIPS/*mrp-l*/MRP-N class.

### Lowest soybean phytate content is solely attributed to combination of two MRP mutations

**Fig 1:**
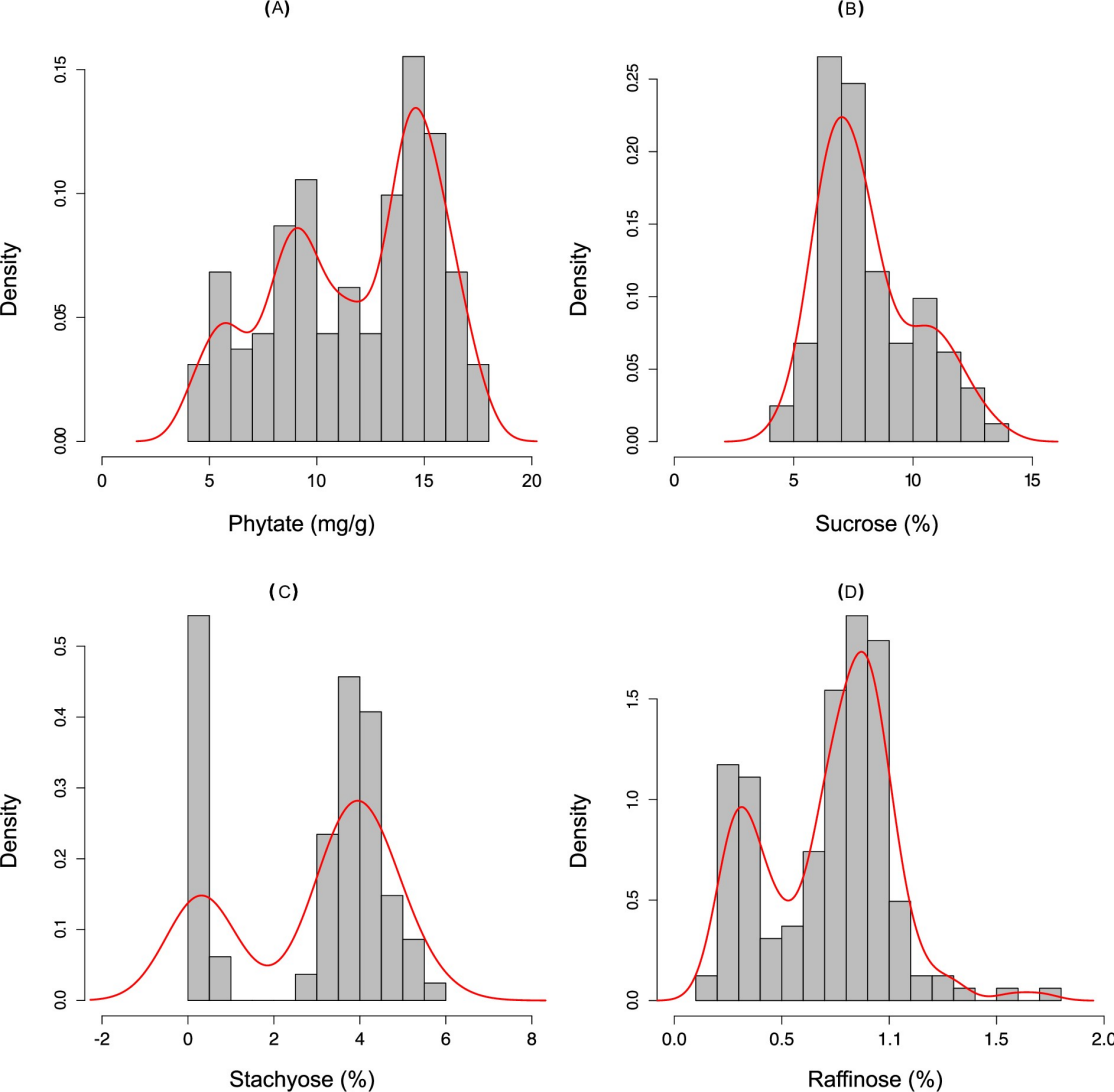
Distribution of seed phytate and oligosaccharide contents in the V99-5089 x CX1834 recombinant inbred population. Density plots represent data from RILs (N = 163) with homozygous alleles at three *lpa* causing mutational sites. (a) Phytate data was collected at the F_8_ generation, whereas (b) sucrose, (c) stachyose, and (d) raffinose data were collected at the F_6_ generation. Gaussian kernel density estimation for each of these datasets is indicated in red. The oligosaccharide (sucrose, raffinose, stachyose) contents are represented as percentage of dry seed weight.

**Fig 2:**
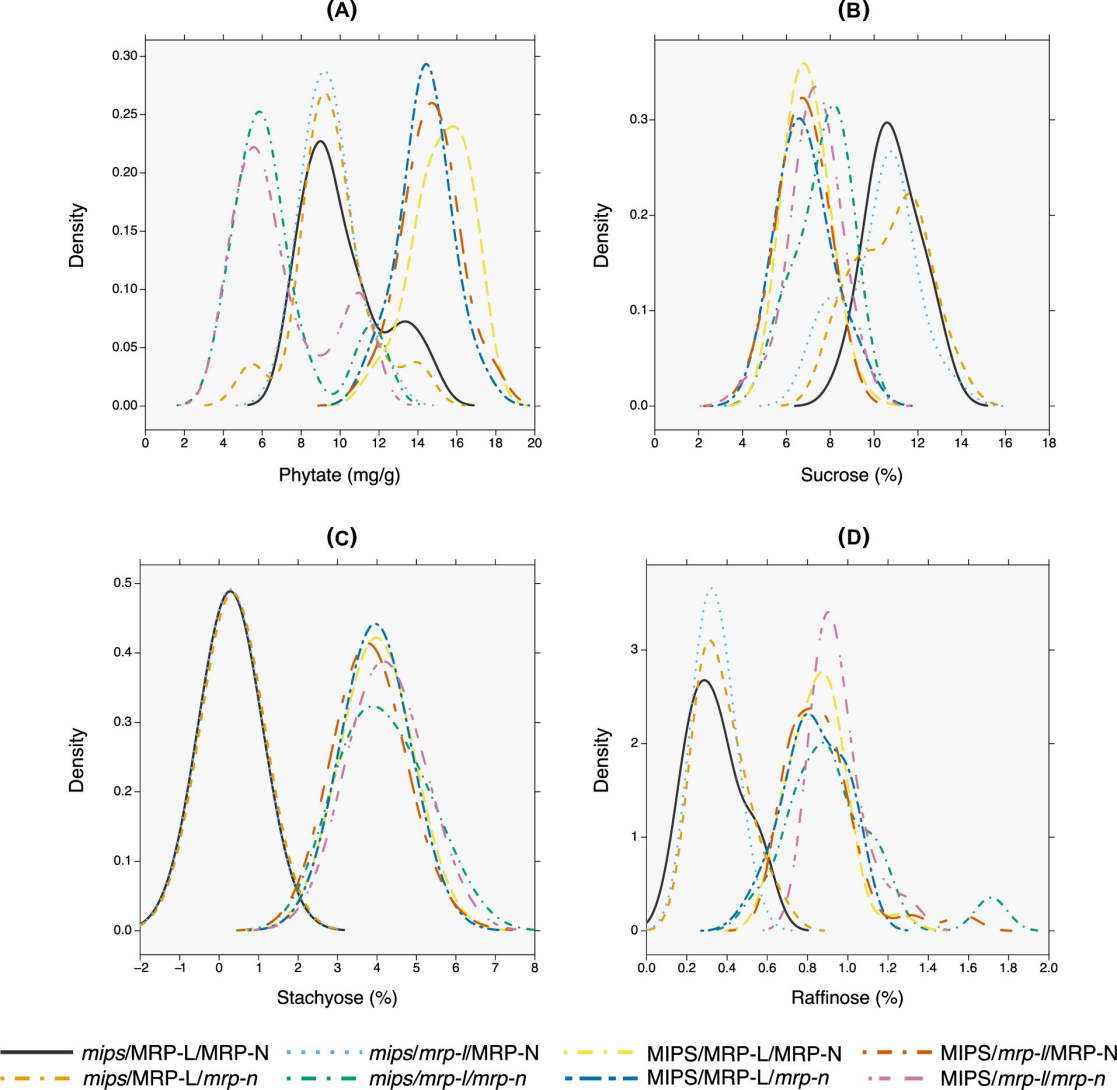
Distribution of seed phytate and oligosaccharide contents of recombinant inbred lines in the eight genotypic classes within the V99-5089 x CX1834 RIL population. Gaussian kernel density estimation for each of these datasets is represented for each genotypic class at the bandwidth of 0.8 for (a) phytate, (b) sucrose, (c) stachyose, and 0.08 for (d) raffinose. The oligosaccharide (sucrose, raffinose, stachyose) contents are represented as percentage of dry seed weight.

The phytate values for the low-phytate parental lines ranged from 8.78 mg g^-1^ for CX1834 to 10.62 mg g^-1^ for V99-5089, as compared with 15.2 mg g^-1^ observed in the homozygous wild-type RILS (MIPS/MRP-L/MRP-N) (**Table 2**). The phytate values for the RILs in this study ranged from 4.01 to 17.83 mg g^-1^, with the overall mean of 11.73 mg g^-1^ (**Table 2**). The genotypic class *mips*/*mrp-l*/*mrp-n* containing all three *lpa* causing mutations showed the lowest mean phytate content of 6.70 mg g^-1^; whereas the genotypic class MIPS/MRP-L/MRP-N without any *lpa* causing mutations showed the highest mean phytate content of 15.22 mg g^-1^ (**Table 2**). The density of the phytate values for all homozygous RIL individuals (N = 163) in this population appears to be multimodal distribution with three peaks located at approximately 5–6 mg g^-1^, 9–10 mg g^-1^, and 14–15 mg g^-1^ (**Fig 1A**). The mean phytate values for the eight genotypic classes formed three statistically different groups (Pairwise Wilcox test p-value < 0.01) with density peaks in close proximity to the three phytate ranges described above (**Fig 2A**). In other words, each phytate peak represented RIL individuals with different combinations of *lpa* causing mutations.

**Table 2:** Mean phytate, sucrose, raffinose, and stachyose contents for the genotypic classes of the V99-5089 x CX1834 population.

Parental lines		Phytate (mg g^-1^)^b^		Sucrose (%)^b^		Raffinose (%)^b^		Stachyose (%)^b^	
V99-5089 (*mips*/MRP-L/MRP-N)		10.62		12.50		0.53		0.25	
CX1834 (MIPS/*mrp-l*/*mrp-n*)		8.78		7.14		0.73		4.48	
Genotypic classes of RILs^a^	N	Mean ± S.D.	Range	Mean ± S.D.	Range	Mean ± S.D.	Range	Mean ± S.D.	Range
*mips/*MRP-L/MRP-N	15	10.09±2.0 **a**	7.65–14.47	10.91±1.04 **a**	8.82–12.78	0.34±0.12 **a**	0.16–0.56	0.30±0.17 **a**	0.13–0.78
*mips/*MRP-L/*mrp-n*	14	9.55±1.97 **a**	5.43–14.0	10.81±1.63 **a**	8.22–13.65	0.37±0.11 **a**	0.25–0.64	0.36±0.18 **a**	0.15–0.67
*mips/mrp-l*/MRP-N	20	9.36±1.27 **a**	7.06–12.43	10.23±1.63 **a**	7.24–13.51	0.33±0.07 **a**	0.22–0.49	0.30±0.13 **a**	0.17–1.53
*mips/mrp-l/mrp-n*	14	6.70±2.40 **b**	4.01–11.96	7.70±1.10 **b**	5.64–9.17	0.94±0.28 **b**	0.55–1.71	4.18±0.90 **b**	2.87–5.92
MIPS/MRP-L/MRP-N	29	15.22±1.4 **c**	12.29–17.24	6.95±0.78 **b**	5.83–9.15	0.85±0.13 **b**	0.63–1.25	4.02±0.50 **b**	3.04–5.10
MIPS/MRP-L/*mrp-n*	24	14.37±1.3 **d**	11.53–17.33	6.89±1.15 **b**	4.96–9.48	0.83±0.15 **b**	0.51–1.07	3.98±0.42 **b**	3.16–4.82
MIPS*/mrp-l/*MRP-N	30	14.97±1.4 **cd**	11.26–17.83	6.75±0.94 **b**	4.66–8.49	0.87±0.19 **b**	0.65–1.57	3.84±0.57 **b**	2.90–5.58
MIPS/*mrp-l/mrp-n*	17	6.94±2.45 **b**	4.14–11.22	7.29±1.07 **b**	4.52–9.27	0.95±0.12 **b**	0.82–1.29	4.25±0.65 **b**	3.16–5.49
**All classes (N = 163)**		**11.73±3.68**	**4.01–17.83**	**8.10±2.02**	**4.52–13.65**	**0.72±0.29**	**0.16–1.71**	**2.89±1.77**	**0.13–5.92**

^a^ Genotypic classes are designated in order of MIPS allele on chr. 11 (LG B1)/MRP-L allele on chr. 19 (LG L)/MRP-N allele on chr. 3 (LG N). Lower case, italicized letters indicate mutant alleles and upper-case letters indicate wild-type alleles.

^b^ Mean and standard deviation (S.D.) for phytate, sucrose, raffinose, and stachyose is calculated from all the lines in each genotypic class. Phytate data was collected at the F_8_ generation, while oligosaccharide data were collected at the F_6_ generation. The oligosaccharide (sucrose, raffinose, stachyose) contents are represented as percentage of dry seed weight. For each trait, genotypic classes with values significantly different from one another are indicated by different letters, as determined with Pairwise Wilcox test to compare genotypic class means at significance level of 0.05 (for phytate) and 0.01 (for oligosaccharides).

RILs from two genotypic classes–*mips/mrp-l/mrp-n* (N = 14, phytate = 6.70 mg g^-1^) and MIPS/*mrp-l*/*mrp-n* (N = 17, phytate = 6.94 mg g^-1^) with lower mean phytate values showed overlapping density peaks around the lowest range (5–6 mg g^-1^) of phytate values (**Table 2; Fig 2A)**. There was no significant difference between phytate values of RIL individuals from these two genotypic classes (**Table 2**). Two mutations in common between these two genotypic classes are the *lpa* causing mutations in MRP genes on chr. 19 (LG L) and chr. 3 (LG N). This suggests that perhaps only MRP gene mutations (*mrp-l/mrp-n*) are enough to achieve the lowest levels of phytate. However, the MIPS/*mrp-l*/*mrp-n* genotypic class showed lower phytate values than the parental line CX-1834 (phytate = 8.78 mg g^-1^) with identical mutation combinations. This difference in the phytate values of the MIPS/*mrp-l*/*mrp-n* genotypic class and the parent CX-1834 could be due to some unidentified genetic contributions from the second parent V99-5089.

Similarly, three genotypic classes–*mips*/MRP-L/MRP-N (N = 15, phytate = 10.09 mg g^-1^), *mips*/MRP-L/*mrp-n* (N = 14, phytate = 9.55 mg g^-1^), and *mips/mrp-l*/MRP-N (N = 20, phytate = 9.36 mg g^-1^) showed overlapping density peaks around midrange (9–10 mg g^-1^) of phytate values (**Table 2; Fig 2A**). One mutation in common between these three genotypic classes is the *mips* mutation on chr. 11 (LG B1). This suggests that *mips* mutation alone could be enough to achieve the midrange of phytate levels and perhaps combining this mutation with either of the *mrp* mutations does not contribute to any further reduction in the phytate levels.

The remaining three genotypic classes–MIPS/MRP-L/MRP-N (N = 29, phytate = 15.22 mg g^-1^), MIPS/MRP-L/mrp-n (N = 24, phytate = 14.37 mg g^-1^), and MIPS/*mrp-l*/MRP-N (N = 30, phytate = 14.97 mg g^-1^) showed overlapping density peaks around 14–15 mg g^-1^ of phytate (**Table 2; Fig 2A**). This represents phytate levels typical of wild-type soybeans. This suggests that wild type MIPS allele is required to confer highest phytate levels, provided either one or both MRP alleles are wild type. In summary, the combination of *mrp-l* and *mrp-n* mutations (like CX-1834 parent) could drop seed phytate content to the lowest levels; whereas the *mips* mutation alone (like V99-5089) could reduce the phytate level but cannot achieve the lowest levels.

### Effects of *mips* mutation on seed oligosaccharides are reversed in the presence of *mrp-l/mrp-n* mutations

The two parents of this population (V99-5089 and CX1834) have contrasting oligosaccharide contents, in particular sucrose and stachyose. V99-5089 is a high-sucrose, low-stachyose line having a mean sucrose at 12.5% and stachyose at 0.25% of the dry seed weight (n = 9 assays). CX1834 has a low-sucrose value, averaging 7.14%, and high-stachyose, at 4.48% of the dry seed weight (n = 7 assays). Raffinose values for two parents were roughly similar (V99-5089 = 0.53% and CX-1834 = 0.73% of dry seed weight) (**Table 2**). The oligosaccharide data for the RIL population (N = 163) was as follows: mean sucrose at 8.10% (range 4.52–13.65%), mean raffinose at 0.72% (range 0.16–1.71%), and mean stachyose at 2.89% (range 0.13–5.92%) of the dry seed weight (**Table 2**).

The density of oligosaccharide (sucrose, stachyose and raffinose) data for all homozygous RIL individuals (N = 163) in the population followed differing distributions: an unimodal distribution for sucrose with peak at 7%, and bimodal distribution for stachyose and raffinose with two peaks at approximately 0.32% and 4.05% for stachyose, and 0.34% and 0.88% for raffinose (**Fig 1B, 1C and 1D**). For each of these oligosaccharides, two statistically different groups were formed (Pairwise Wilcox test p-value < 0.01), comprised of five (indicated by group letter ‘b’ in **Table 2**) and three (indicated by group letter ‘a’ in **Table 2**) genotypic classes each (**Table 2, Fig 2**). The genotypic classes within each group showed overlapping peaks for all three oligosaccharides (**Table 2, Fig 2**).

The first group included five genotypic classes (indicated by group letter ‘b’ in sucrose, raffinose and stachyose columns in **Table 2**), with no mutation common in all five genotypes. This group showed lower sucrose (~7%), and higher stachyose (~ 4%) and raffinose (~0.8%) contents compared to that of the second group (sucrose >10%, stachyose ~0.3%, and raffinose ~0.3% of dry seed weight) (**Fig 2B, 2C and 2D**). The second group included three genotypic classes–*mips*/MRP-L/MRP-N, *mips*/MRP-L/*mrp-n*, and *mips*/*mrp-l/*MRP-N. These classes had *mips* mutation in common, suggesting that *mips* mutation is required to achieve the higher sucrose, lower stachyose and raffinose levels; provided either one or both MRP alleles are wild type. In other words, *mrp-l* and/or *mrp-n* alleles alone do not affect oligosaccharide levels in the soybean seeds.

Interestingly, oligosaccharide values for the *mips/mrp-l/mrp-n* class were unexpected (**Table 2**). Since this genotypic class has all three mutations, it was expected to have low-phytate as well as high-sucrose and low-stachyose (low-phytate being conferred by V99-5089 and CX1834, and low-stachyose being conferred by V99-5089). Instead, the *mips*/*mrp-l*/*mrp-n* class showed lower sucrose, high-stachyose and raffinose contents (**Table 2**). Due to this unexpected outcome, we evaluated phenotypic data for *mips*/*mrp-l*/*mrp-n* genotypic class from subsequent generations (phytate data from the F_9_ and F_10_ generations and the oligosaccharide data from the F_8_ and F_10_ generations). For all subsequent generations, it was confirmed that combining all three *lpa* causing mutations results in low-sucrose, high-stachyose, and high-raffinose contents in soybean (**Table 3**). This suggests that the high-sucrose and low-stachyose, and low-raffinose phenotype conferred by the *mips* mutation could be reversed when both *mrp-l* and *mrp-n* mutations are in the genetic background.

**Table 3:** Recombinant inbred lines in the *mips/mrp-l/mrp-n* genotypic class of the V99-5089 x CX1834 population.

RIL IDs ^a^	Phytate (mg/g)			Sucrose (%)			Stachyose (%)			Raffinose (%)		
	F_8_	F_9_	F_10_	F_6_	F_8_	F_10_	F_6_	F_8_	F_10_	F_6_	F_8_	F_10_
819	5.09	3.96	4.97	8.09	8.06	11.28	3.76	4.20	6.67	0.90	0.77	1.14
821	ND	5.90	5.59	8.09	5.89	7.85	3.22	2.87	6.71	0.62	0.55	1.40
831	6.53	6.26	6.52	8.29	8.23	7.05	2.78	4.09	6.10	0.68	0.89	1.47
833	5.43	5.12	6.19	7.56	8.27	5.74	3.85	4.61	5.33	0.66	0.88	0.99
853	ND	5.35	4.43	7.27	6.96	6.84	3.42	3.50	4.97	0.80	0.72	1.07
859	ND	6.1	4.92	6.86	6.43	6.35	3.04	3.18	5.48	0.68	0.71	1.03
866	7.85	7.59	5.19	7.96	7.83	6.52	3.32	5.12	5.69	0.70	0.80	1.10
884	4.5	6.52	2.41	8.23	9.17	10.45	3.32	3.48	5.43	0.84	1.10	1.14
886	4.6	5.43	4.50	7.68	8.81	6.75	4.03	5.25	6.40	0.81	0.94	0.90
903	5.4	5.59	6.15	ND	7.35	5.30	ND	3.50	5.26	ND	0.96	1.48
887	ND	ND	ND	ND	5.64	ND	ND	5.92	ND	ND	1.19	ND
959	7.89	8.67	3.90	9.88	8.23	8.23	2.63	5.09	5.09	0.62	0.89	0.89
998	5.6	6.87	3.33	7.43	8.79	6.70	3.30	3.51	4.48	0.88	1.71	0.72
1006	4.67	5.58	ND	7.98	8.16	ND	3.64	4.16	ND	0.98	1.10	ND
**Mean**	**5.76**	**6.07**	**4.84**	**7.94**	**7.70**	**7.42**	**3.36**	**4.18**	**5.63**	**0.76**	**0.94**	**1.11**

^a^ Oligosaccharide content data were collected by HPLC for the F_6_, F_8_, and F_10_ generations. Phytate data were collected for the F_8_, F_9_, and F_10_ generations. All fourteen lines were confirmed to each have all three mutations. Abbreviation: ND means ‘not determined’. The oligosaccharide (sucrose, raffinose, stachyose) contents are represented as percentage of dry seed weight.

## Discussion

The current study was focused on understanding the impact of combining *lpa* causing mutations in a MIPS gene (in line V99-5089) and two MRP genes (in line CX1834) on phytate and oligosaccharides in the soybean RIL population from a cross of V99-5089 x CX1834. Three SNP markers were designed to detect mutant alleles for each of the three genes that cause the low-phytate phenotype in soybean. These markers allowed for easy genotyping of the recombinant inbred population and should be more effective than phenotypic selection, or even selection using tightly linked SSRs (Simple Sequence Repeats). These perfect markers could be a valuable asset to the soybean breeding programs for marker-assisted selection of phytate and oligosaccharide content.

Eight genotypic classes of the RILs, comprised of all possible combinations of mutant and wild type alleles of the three genes (*mips* or MIPS, *mrp-l* or MRP-L, and *mrp-n* or MRP-N), were identified using these SNP markers and were further evaluated for the seed phenotypes in their advanced generations (from F_6_ to F_10_). With reference to sucrose and raffinosachharides, these eight genotypic classes formed two groups. The first group, comprised of RIL individuals from *mips*/MRP-L/MRP-N, *mips*/*mrp-l/*MRP-N, and *mips*/MRP-L/*mrp-n* classes, showed moderately reduced phytate, high-sucrose, low-stachyose, and low-raffinose phenotypes, similar to the V99-5089 *lpa* parent. The second group, comprised of RIL individuals from remaining five genotypic classes (MIPS/MRP-L/MRP-N, MIPS/MRP-L/*mrp-n*, MIPS/*mrp-l*/MRP-N, MIPS/*mrp-l*/*mrp-n*, and *mips/mrp-l*/*mrp-n*) showed normal-sucrose, high-stachyose, and high-raffinose phenotypes, similar to the CX-1834 parent. Two contrasting trends were noticed for the phytate phenotype within this second group. The genotypic classes MIPS/*mrp-l*/*mrp-n* and *mips/mrp-l*/*mrp-n* showed similar, more substantially reduced phytate typical of the CX-1834 parent, whereas the remaining genotypic classes within this group displayed wild-type levels of seed phytate. Overall, combining three mutations resulted in lowest phytate levels. This suggests, although both *mips* and *mrp-l/mrp-n* mutations can independently result in reduced seed phytate, *mrp-l/mrp-n* mutations show dominance over *mips* mutation in regulating seed phytate levels. This research also provided evidence for the reversal of the seed oligosaccharide phenotype of *mips* mutation in the presence of *mrp-l/mrp-n* mutations. These findings confirm the interaction of MIPS and MRP genes in regulating phytate and RFO biosynthesis pathways in soybean.

The MIPS and MRP gene interactions were previously studied using soybean breeding lines originating from V03-5901 (*mips*) x 04-05N32 (*mrp-l/mrp-n*) cross [48]. Compared to this study, the triple mutants from Averitt et al. [48] showed higher sucrose (8.25%), lower raffinose (0.86%) and lower stachyose (3.69%) levels; however, only phytate and raffinose levels were significantly different from the *mips* mutant. Despite the fact that the lowest phytate level (ranging from 0.227–4.864 mg g^-1^) was attained for triple mutant in Averitt et al. [48], it was not significantly different from *mrp-l*/*mrp-n* mutant, and this observation is consistent with the current study. Different seed phytate levels of triple mutants in these two studies could be due to dissimilarities in the genetic backgrounds of the parental lines used in two studies. Importantly though, Averitt et al. [39] observed a similar effect of homozygosity for *mrp-l/mrp-n* (referred to as *lpa1* and *lpa2*) on the *mips* low-stachyose phenotype. As observed here, Averitt et al. [39] reported what appeared to be a trend for normal, not-reduced levels of stachyose in homozygous *mips* lines that were also homozygous *mrp-l/mrp-n*.

Another study with a low-phytate *Phaseolus* (common bean) mutant line *lpa1* (280–10), with mutations in two MRP genes encoding ABC transporters (most likely orthologous to soybean *mrp-l* and *mrp-n* mutations) was shown to have 90% and 25% reductions in the phytate and RFOs contents, respectively [49]. Panzeri et al. [33] did not notice any accumulation of phytate in the cytoplasm even though there was no obstruction in the phytate biosynthesis pathway, suggesting that a potential negative feedback regulation results in degradation of phytate in the cytoplasm to produce *myo*-inositol that is subsequently used in the RFO biosynthesis pathway. It is possible that such a negative feedback mechanism may also exist in soybean, where highly unstable free phytate in the cytoplasm could breakdown into its precursors, free *myo*-inositol and lower-order phosphates, ultimately reducing phytate levels.

**Fig 3:**
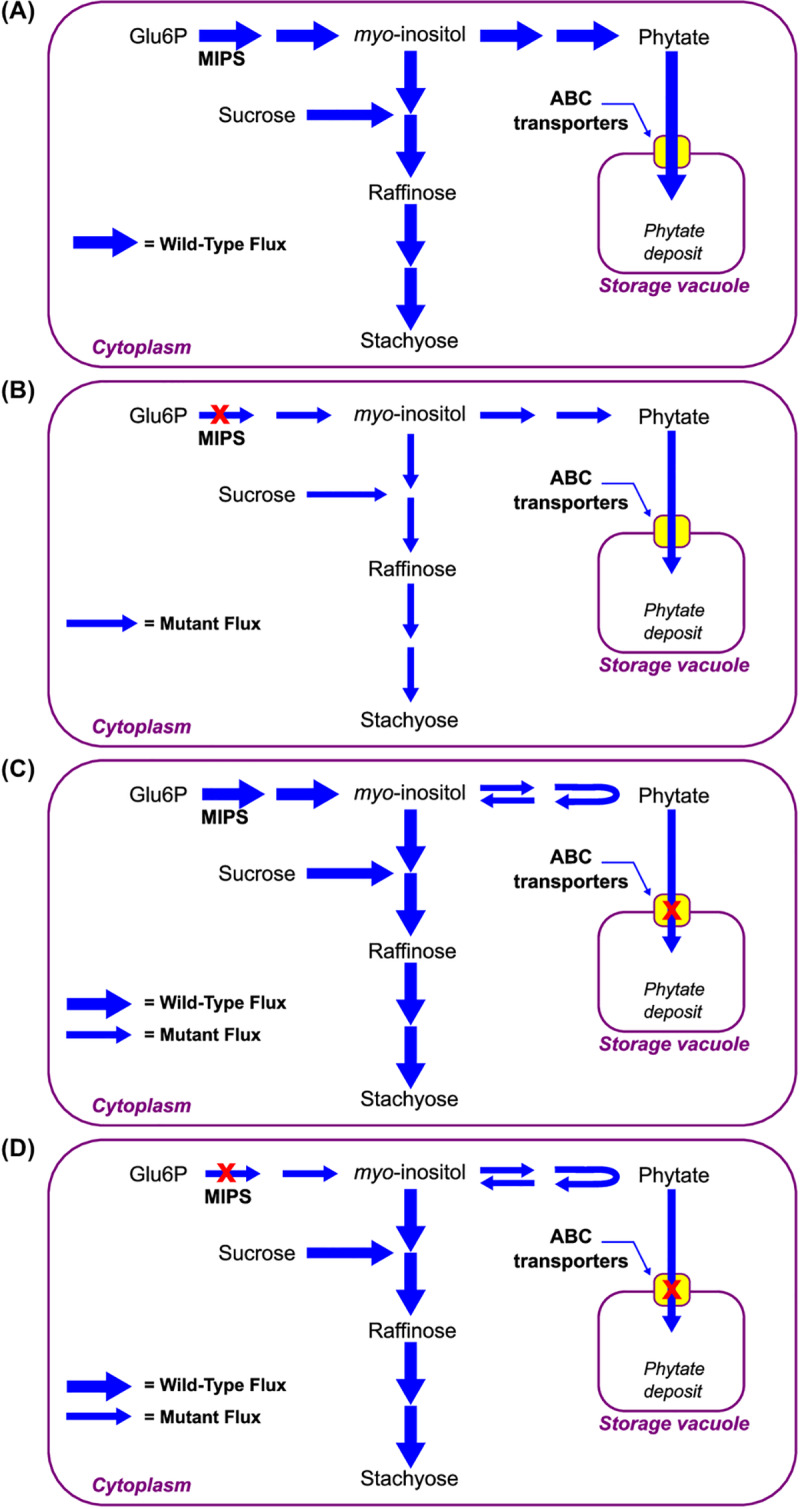
Interaction between the MIPS and MRP genes in V99-5089 x CX1834 recombinant inbred population. (A) Proposed model of MIPS and MRP gene interactions for seed phytate and oligosaccharide phenotypes in soybean. This model is applicable to three genotypic classes—MIPS/MRP-L/MRP-N, MIPS/*mrp-l*/MRP-N, and MIPS/MRP-L/*mrp-n*. (B) In V99-5089, MIPS is defective, therefore *myo*-inositol supply is reduced leading to low-phytate, low-RFOs, accumulation of unutilized sucrose. This model is also applicable to three genotypic classes—*mips*/MRP-L/MRP-N, *mips*/*mrp-l*/MRP-N, and *mips*/MRP-L/*mrp-n*. (C) In CX1834, phytic acid transport into the protein storage vacuoles is reduced due to mutation in MRP genes, causing low-phytate. The RFO and sucrose levels are unaffected in these mutants. This model is also applicable to MIPS/*mrp-l/mrp-n* genotypic class. (D) When three mutations are combined (as in *mips/mrp-l/mrp-n* genotypic class), lower levels of myo-inositol and phytate are being produced as a result of *mips* mutations. As ABC transporters are impaired by *mrp-l/mrp-n* mutations, cytoplasmic phytate gets broken down into its precursors, *myo*-inositol and lower-order intermediates and phosphates. This replenishes the levels of cytosolic *myo*-inositol, which is then diverted into the RFO pathway for biosynthesis of raffinose and stachyose. Therefore, oligosaccharide levels in the triple mutant appear to be unaffected.

A possible negative feedback mechanism and the interconnectedness between the phytate and RFO biosynthesis pathways could explain the observed seed phenotypes in the current study (**Fig 3A**). *Myo*-inositol–a common intermediate between the phytate and RFO biosynthesis pathways is utilized as a substrate for formation of galactinol in the first step of the RFO biosynthesis pathway. In this pathway, sucrose (the end product of starch metabolism) is utilized for synthesis of raffinose, which is then utilized for synthesis of stachyose. *Myo*-inositol is synthesized from inositol-3-phosphate in the second step of the phytate biosynthesis pathway. It is subsequently phosphorylated in multiple steps to form phytate [50–52]. MRP gene-encoded ABC transporters are involved in phytate transport from cytoplasm to protein storage vacuoles, where it is stored as a mineral nutrient reserve for seed germination [35, 53]. Both MRP mutations must coexist to impair ABC transporters and prevent phytate translocation [7, 32, 33, 35, 36, 53, 54]. This scenario (**Fig 3A**) applies to MIPS/MRP-L/MRP-N, MIPS/*mrp-l*/MRP-N, and MIPS/MRP-L/*mrp-n* genotypic classes with a functional MIPS enzyme and ABC transporters.

When the *mips* mutation limits the formation of *myo*-inositol precursor (inositol-3-phosphate), reduced levels of *myo*-inositol are produced, which is then shared between the two biosynthesis pathways. A lower level of myo-inositol explains the reduction in the phytate, raffinose and stachyose (biosynthesis products) and the increase in sucrose (substrate) (as in V99-5089 parent and *mips*/MRP-L/MRP-N class) (**Fig 3B**). Similar trends were also noted for *mips*/*mrp-l*/MRP-N and *mips*/MRP-L/*mrp-n* genotypic classes with functional ABC transporters. However, when *mrp-l* and *mrp-n* mutations coexist, only transport of phytate is compromised, not the phytate and RFO biosynthesis pathways (**Fig 3C**). Theoretically, the *myo*-inositol supply for raffinosaccharide levels is not affected in these mutants, since the sucrose, raffinose and stachyose levels are maintained (as in CX-1834 parent and MIPS/*mrp-l*/*mrp-n* genotypic class). While both MIPS and MRP gene mutations could only reduce, but not completely eliminate seed phytate level, the extent of reduction achieved with *mrp-l/mrp-n* mutations (8.78 mg g^-1^ phytate in CX-1834) was greater than with *mips* mutation (10.62 mg g^-1^ phytate in V99-5089).

When three mutations are combined (*mips/mrp-l/mrp-n*), we hypothesize that the reduced levels of *myo*-inositol due to *mips* mutations are in part balanced by *myo*-inositol released from the breakdown of cytoplasmic phytate build-up resulting from the block in its transport to storage vacuoles conditioned by the *mrp-l/mrp-n* mutations (**Fig 3D**). It appears that this free *myo*-inositol is then being utilized for RFO biosynthesis. This could explain why sucrose, stachyose, and raffinose levels are maintained in the *mips/mrp-l/mrp-n* genotypic class.

Remarkably, the *mips/mrp-l/mrp-n* genotypic class has shown greater reduction in seed phytate as compared to the parental lines. Although lines with reduced phytate level are favored in breeding programs, the major challenge is to overcome the low seedling emergence [55, 56]. Four scenarios (**Fig 3**) described above do not clearly indicate the relationship of these mutations with the seed emergence phenotype, although the storage form of phytate could be the key to uncover this relationship. Other mechanisms may also be interfering at different levels to regulate seed phenotypes in *lpa* mutants. For example, several transcription factors and biological processes were found differentially expressed in the developing seeds of *mips/mrp-l/mrp-n* and MIPS/MRP-L/MRP-N lines [57, 58]. Similarly, near isogenic lines with *mrp-l/mrp-n* and MRP-L/MRP-N genotypes were shown to differ in seed metabolites known to be associated with germination process [59]. Epigenetic heritable regulation via paramutagenic interaction between ZmMRP4 alleles (*lpa241* mutation) in maize was also reported in low-phytate maize [27]. Further research is needed to understand the multifaceted regulation of seed germination in low-phytate lines.

In conclusion, genotypic and phenotypic evaluations of the V99-5089 (*mips* mutant line) x CX1834 (*mrp-l/mrp-n* mutant line) recombinant inbred population have revealed the merits of this population in understanding the effects of combining three *lpa* mutations on seed phytate and oligosaccharides on a population-wide level. This population could be a valuable genetic resource to study the *lpa* trait in soybean and its implications on seed emergence and yield, to overcome the challenges of producing high yielding commercial low-phytate soybean.
